# Development and psychometric properties of a new brief scale for subjective personal agency (SPA-5) in people with schizophrenia

**DOI:** 10.1017/S2045796020000256

**Published:** 2020-04-10

**Authors:** Sosei Yamaguchi, Takuma Shiozawa, Asami Matsunaga, Peter Bernick, Utako Sawada, Ayano Taneda, Takahiro Osumi, Chiyo Fujii

**Affiliations:** 1 Department of Community Mental Health & Law, National Institute of Mental Health, National Center of Neurology and Psychiatry, Kodaira, Tokyo 187-8553, Japan; 2 Student Accessibility Office, Nagasaki University, 1-14 Bunkyo-machi, Nagasaki 852-8521, Japan; 3 Department of Psychiatric Nursing, Graduate School of Medicine, The University of Tokyo, 7-3-1 Hongo, Bunkyo-ku, Tokyo 113-8654, Japan; 4 Faculty of Health and Social Services, Kanagawa University of Human Services, 1-10-1 Heisei-cho, Yokosuka, 238-8522, Japan

**Keywords:** Assertive community treatment, personal agency, scale development, schizophrenia

## Abstract

**Aims:**

Personal agency is a variable which potentially facilitates personal recovery in people with serious mental illness. This study aimed to develop a new brief measure for subjective personal agency that can be completed by people with serious mental illness.

**Methods:**

Two focus group interviews were first conducted with 11 people with schizophrenia to understand the fundamental components of subjective personal agency for people with serious mental illness living in the community. One group comprised six people with schizophrenia living in the community, while the other consisted of five people with schizophrenia working as peer-support workers. We then developed scale items through collaboration with people with schizophrenia and qualitative analysis (stage 1). A cross-sectional survey was then conducted to test the psychometric properties of the new scale among service users with schizophrenia in 18 assertive community treatment teams (stage 2). Factor validity was tested via exploratory factor analysis (EFA) and confirmatory factor analysis (CFA). We evaluated convergent validity with the Boston University Empowerment Scale (BUES), divergent validity with the global assessment of functioning (GAF), internal consistency, and test–retest reliability.

**Results:**

Seven items were included in the scale at stage 1. In stage 2, 195 participants completed this scale. EFA revealed a one-factor model with five items. CFA indicated good model fit (*χ*^2^ statistics [CMIN] = 8.445, df = 5 (CMIN/df = 1.689), *p* = 0.133, comparative fit index = 0.974, Tucker–Lewis fit index = 0.949, root mean square error of approximation = 0.077 and standardised root mean squared residual = 0.042). The new scale was significantly correlated with total BUES score (*r* = 0.526, *p* < 0.001), but not with GAF score. Cronbach's *α* for internal consistency was 0.79, and intra-class correlation coefficient for test–retest reliability was 0.70.

**Conclusion:**

We developed a new, five-item Subjective Personal Agency scale (SPA-5) that can be completed by people with serious mental illness. Further studies are needed to confirm the results outside Japan.

## Introduction

Personal agency has come to be seen as an important concept that encompasses the broad range of activities through which people with severe mental illness, such as schizophrenia, take an active role in making meaning of their lives (Lysaker and Leonhardt, 2012). While there are numerous definitions of personal agency, recently in the mental health field, it has been briefly defined as ‘*what people can do on their own* (p. 157)’ (Bellack and Drapalski, 2012).

Of course, ‘agency’ itself is not a new concept. There are two main tracks that reflect how the concept has developed in the fields of social science and neuroscience, respectively. In social science, for example, agency is defined as ‘*what a person is free to do and achieve in pursuit of whatever goals or values he or she regards as important* (p. 203)’(Sen, 1985), or ‘*an actor's or group's ability to make purposeful choices* (p. 3)’ (Samman and Santos, 2009). Studies have also suggested relevant constructs of agency, including subjective freedom and ownership of one's own life, and have identified agency as being particularly proximate to empowerment and well-being (Sen, 1985; Alkire, 2005; Ibrahim and Alkire, 2007; Samman and Santos, 2009). Interestingly, these constructs, which were initially found in studies conducted in Western countries, have also been observed in Japan (Ito and Akimoto, 2015).

On the other hand, cognitive neuroscience studies have approached the consciousness of oneself as an immediate subject of experience, which is referred to as the ‘minimal self’ (Gallagher, 2000). Following this track, the sense of agency is defined as ‘*the sense that I am the initiator or source of the action* (p. 16)’ (Gallagher, 2000). Researchers in this field often focus on voluntary motor actions, rather than the degree of freedom or goal achievement in one's life in the community or broader society. In fact, a theoretical account of the mechanism of the sense of agency incorporates a classic model of motor control. This model proposes that the sense of agency can emerge when a prediction of sensory consequence that is implicitly produced based on an efference copy of motor commands matches the actual sensory consequence (Frith *et al*., 2000). Multiple studies have presented empirical evidence supporting the possibility that an abnormal predictive process underlies the disruption of the sense of agency in individuals with schizophrenia (Blakemore *et al*., 2000; Ford *et al*., 2007; Voss *et al*., 2010).

While the cognitive neuroscience approach offers important implications for the mechanism of a deficit in the sense of agency in serious mental illness, the social science concept of (personal) agency appears to better fit the community mental health and personal recovery contexts (Lysaker and Leonhardt, 2012; Ciftci *et al*., 2015). Over the past half century, an increasing number of people with serious mental illness, such as schizophrenia, are living in the community as a result of deinstitutionalisation (Kunitoh, 2013). At the same time, the paradigm of personal recovery, which has been driven by service users and which refers to the process whereby people achieve a meaningful life, has become a worldwide movement (Deegan, 1996; Davidson and Roe, 2007). Systematic reviews have identified the core concepts of personal recovery, which include connectedness, hope and optimism, identity, meaning, empowerment and person-centeredness (Leamy *et al*., 2011; Ellison *et al*., 2018; van Weeghel *et al*., 2019). These reviews also found that personal agency in the community life of people with serious mental illness is a key factor that contributes to the individual personal recovery process (Wood and Alsawy, 2018; van Weeghel *et al*., 2019). In this way, the importance of personal agency has recently re-emerged in connection with research into the components of personal recovery (Lysaker and Leonhardt, 2012). Indeed, both personal agency and personal recovery require that service users have subjective views of their own lives, even though these individuals have serious mental illness that may affect the objective agency involved in controlling one's actions (Deegan, 1996; Davidson and Roe, 2007; Leamy *et al*., 2011; Ellison *et al*., 2018; Wood and Alsawy, 2018; van Weeghel *et al*., 2019). In addition, subjective personal agency appears to be related to subjective ownership, where service users feel empowered and believe that they can recover (Wood and Alsawy, 2018).

Given that personal agency is recognised as a factor that facilitates personal recovery (van Weeghel *et al*., 2019), measuring agency is useful for both research and clinical practice. However, few tools have been developed to directly assess subjective personal agency in people with serious mental illness, such as schizophrenia (Tapal *et al*., 2017). In addition, existing scales for empowerment, which is conceptually related to personal agency, may not have been developed in collaboration with people with serious mental illness; these scales also often have a large number of response items, which can be a barrier to completion for the target population (McCabe *et al*., 2007; Barr *et al*., 2015). These are major issues in the context of developing a patient-reported measure, given that service user involvement in research and the clinical usefulness of a brief scale have been internationally emphasised (Reininghaus and Priebe, 2012; Staniszewska *et al*., 2012; Wykes, 2014; Wiering *et al*., 2017). The present study therefore aimed to develop a new, brief, patient-reported measure of subjective personal agency, based on service users' views and through collaboration with people with schizophrenia.

## Methods

### Overall design

This study was conducted in two stages. The first stage consisted of focus group interviews with people with schizophrenia, in order to create initial scale items. The second stage was a cross-sectional survey of assertive community treatment (ACT) service users with schizophrenia to test the scale's psychometric properties.

### Stage 1: focus group interviews and item development process

To create initial scale items, we conducted two 2 h focus group interviews. One group comprised six people with schizophrenia living in the community, while the other consisted of five people with schizophrenia working as peer-support workers. This study was approved by the Research Ethics Committee at the National Center of Neurology and Psychiatry (no. A2016-066). Participants were recruited from across Japan via the e-mail newsletter of a non-profit organisation, the Community Mental Health & Welfare Bonding Organization, which connects service users, service providers and researchers in collaborative facilities and recovery-oriented services, and develops user-led events. Each focus group interview featured group facilitators who were other peer-support workers with schizophrenia. Prior to the interviews, we developed an interview guide, which contained the time schedule/flow of the interviews, the aims of the study, a very brief definition of personal agency and a list of interview questions.

In the focus group interviews, we briefly explained personal recovery and personal agency, and asked when and how participants experienced subjective personal agency in their daily lives based on the interview guide. Interviews were audio recorded and transcribed. Four authors (SY, AM, US, AT) qualitatively analysed interview data via the qualitative descriptive method (Miles *et al*., 2014). In the initial coding process, at least two authors independently extracted tentative codes from the transcribed interview data. After initial coding, the four authors jointly analysed and thoroughly discussed subcategory development, resulting in the classification of similar codes into 11 subcategories. This inductive coding was conducted over several iterations. Finally, the 11 subcategories were classified into five key categories of personal agency (Table 1). Based on the categories that were generated by the qualitative analysis, and after considering other relevant studies (Sen, 1985; Alkire, 2005; Ibrahim and Alkire, 2007; Samman and Santos, 2009; Ito and Akimoto, 2015; Tapal *et al*., 2017; Wood and Alsawy, 2018; van Weeghel *et al*., 2019), we developed an initial seven-item version of the Subjective Personal Agency scale (SPA).

**Table 1.** Item development and content analysis for group interviews on subjective personal agency in people with schizophrenia

Scale item		Category	Category definition	Sub-category
1	I think for myself and make my own life decisions	Making decisions based on one's own ideas/thinking.	Making one's own decisions regardless of the situation, and choosing from among the options which someone presents or the individual themselves perceives as being available.	Making decisions without being influenced by one's surroundings. Selecting one option from those presented or those perceived as possible.
2	I have an idea of what I want to do and/or how I want to be.	Having a concrete image of ‘what one wants to be’, and action taken towards its realisation.	Knowing what one hopes and what one wants to do, and taking action towards realising it.	Being cognisant of/having an image of ‘what one wants to be’. Taking action to realise ‘what one wants to be’.
3	I am taking concrete steps to realise what I want to do and/or how I want to be.			
4	I express myself in a way that values my own personal style.	Expressing personal values and one's own style.	Using one's own way of talking about one's personal values and opinions in order to express oneself, regardless of surroundings or others' expectations.	Using one's own style. Expressing personal values and one's own style.
5	I am able to express my thoughts and feelings in my own words.			
6	My choices in lifestyle (e.g. how I use money or time, or my daily life/work) are limited due to things like illness or life circumstances.	No restrictions on choices as a result of one's own circumstances.	Life choices (e.g. use of money or time, or work) are not restricted by illness-related aspects, such as symptoms, overall condition, medication and therapeutic environment, or by general aspects of community life, such as financial issues.	No restrictions on own choices due to symptoms. No restrictions on own choices due to medication or therapeutic environment. No restrictions on own choices due to financial issues.
7	I give up on doing what I want to do because of my own or others' assumptions that I can't do those things.	No restrictions on actions as a result of presumptions on the part of oneself or someone close.	Actions desired to be taken are not restricted due to presumptions – either one's own or those of someone close – that the person cannot do certain things.	No restrictions on own actions due to one's own or others' presumptions. Having the freedom to do nothing.

After initial item development, all focus group participants verified the content of each category, and group facilitators confirmed the category distribution and fit of the seven items, as well as whether item wording was applicable and understandable to people with schizophrenia. In addition, we asked five ACT service users and five ACT staff members which type of scale (visual analogue or Likert-type) was preferable. Based on their input and discussion among the authors and the focus group interview facilitators, we decided to employ a five-point Likert scale for SPA which uses a range of 1 (strongly disagree) to 5 (strongly agree) for each item (online Supplementary Table S1). Finally, nine service users from two ACT teams checked the wording of each item. They were also able to complete the scale in <5 min. It should be noted that the English version of the scale included here is a translation of the Japanese version that was actually used in this study. The English version was developed through extensive discussion and revision by project members familiar with both languages, as well as by back-translation, and it is believed to be essentially equivalent in meaning to the Japanese version.

### Stage 2: evaluating psychometric properties

#### Setting and participants

We conducted a cross-sectional questionnaire survey with ACT teams across Japan to assess the factor structure, cross-validity, convergent validity, internal consistency and test–retest reliability of the new scale. We recruited 20 ACT teams that were registered in the Japan Assertive Community Treatment Network Association and that underwent regular fidelity assessments. A total of 18 ACT teams participated in the study (one declined to participate due to a lack of staff for research work, and another actually closed its ACT service agency). In each participating ACT team, trained staff members recruited a maximum of approximately 15 service users with schizophrenia between 1 January and 31 March 2018. Eligibility criteria were as follows: (1) diagnosed with schizophrenia; (2) use of ACT services in December 2017; (3) aged 20 years or older; and (4) having the capacity to consent to participate in the study. Trained ACT staff members then visited individual service users' homes, explained the study to potential participants and obtained consent to participate. This study was approved by the Research Ethics Committee at the National Center of Neurology and Psychiatry (no. A2017-063).

#### Factor validity

We conducted both exploratory factor analysis (EFA) and confirmatory factor analysis (CFA) to confirm the factor structure and cross-validity of the SPA. We selected the samples for EFA and CFA using random sampling with a ratio of 4:6 at the cluster level, as CFA requires a larger sample compared to EFA (DeCoster, 1998), while factor analysis generally requires at least ten observations per item (Comrey and Lee, 1992). EFA with oblimin rotation was conducted and the number of factors was determined based on scree plots. Items were extracted when they loaded ⩾0.4 and showed significant loading on the factor. We then conducted CFA to test the fit of the model with the data, using the *χ*^2^ statistic (CMIN), comparative fit index (CFI), Tucker–Lewis fit index (TLI), root mean square error of approximation (RMSEA) and standardised root mean squared residuals (SRMR). According to the COnsensus-based Standards for the selection of health Measurement INstruments (COSMIN) guidelines for systematic reviews (Prinsen *et al*., 2018), acceptable model fit values are as follows: CFI>0.95, TLI>0.95, RMSEA<0.06 or SRMR<0.08. Finally, we conducted a Monte Carlo simulation analysis with 5000 repetitions for the CFA sample to address sampling bias. The analysis computed probabilities for the number of simulated replication values for CMIN, RMSEA and SRMR that exceeded the original CFA values corresponding to each index (Muthén and Muthén, 1998–2017). All analyses in this study were conducted using Stata version 15 and Mplus version 8.

#### Convergent and divergent validity

We used the Boston University Empowerment Scale (BUES) to assess convergent validity (Rogers *et al*., 1997). A Japanese version of the BUES exists, and its convergent validity, internal consistency and test–retest reliability have been confirmed (Hata *et al*., 2003). We also used the global assessment of functioning (GAF) to assess overall functioning in participants and examine divergent validity (APA, 1994). Each participant's case manager provided a GAF rating. Pearson's correlation coefficients were computed for convergent validity and divergent validity.

#### Reliability

Cronbach's *α* was calculated to determine internal consistency. Test–retest reliability was assessed using intra-class correlation coefficients (ICC). For test–retest reliability, participants in two ACT teams (*n* = 23) completed the SPA a second time, 2 weeks after initial administration.

## Results

### Study participants

During the recruitment period, ACT staff members visited 280 eligible service users. Of these, 252 received an explanation of the study, and 197 voluntarily consented to participate. Two participants did not complete the questionnaire after initial consent, with the result that data from 195 participants were included in the analysis (online Supplementary Fig. S1). Table 2 lists the characteristics of the participants. Approximately 45% were female, and mean age was 48.59 (s.d. = 11.85). Half of the participants had graduated from high school. The mean GAF score was 41.05 (s.d. = 12.38).

**Table 2.** Characteristics of participants

	*n* = 195	
Sex, *n* (%)		
Female	88	45.13
Male	107	54.87
Age, mean (s.d.)	48.59	11.85
Highest level of school completed, *n* (%)		
Middle (junior high) school	43	22.05
High school	98	50.26
Technical college	9	4.62
Junior college	9	4.62
University (undergraduate degree)	35	17.95
University (graduate degree)	1	0.51
Marital status, *n* (%)		
Never married	159	81.54
Married	9	4.62
Divorced	27	13.85
Living situation, *n* (%)		
Living with family	69	35.38
Living alone	83	42.56
Residential facility	43	22.05
Employment, *n* (%)		
Currently employed	19	9.74
Hospitalisation in past 12 months, *n* (%)		
Have been hospitalised	41	21.03
Global assessment of functioning, mean (s.d.)	41.05	12.38

### Factor analysis

**Fig. 1.**
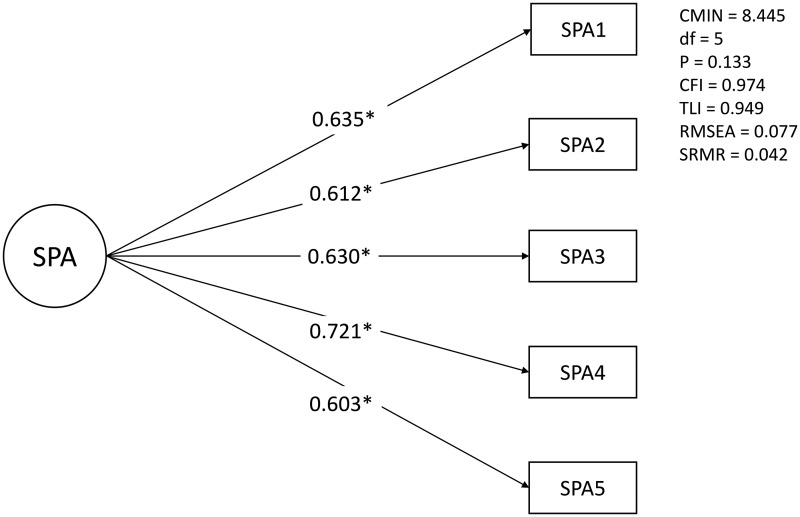
Results of confirmatory factor analysis.

Random sampling was used to select 78 participants from eight ACT teams for EFA (Table 3). The resulting scree plot indicated a borderline two-factor *v*. one-factor model for the SPA (online Supplementary Fig. S2). In the two-factor model, all items loaded at more than 0.4. However, the second factor consisted of only two items (nos. 6 and 7). When a factor has only two items, a high correlation (*r* > 0.6) between the items is needed (Gie Yong and Pearce, 2013). Since the correlation coefficient (*r*) between these items was 0.518, we rejected the two-factor model. The one-factor model also excluded items 6 and 7 due to low factor loading (<0.4), and included the five items (nos. 1–5) which achieved significant factor loading (>0.4). We used this one-factor model consisting of these five items to create the SPA-5. CFA for the SPA-5 was conducted with 117 participants from ten ACT teams. The resulting analysis was as follows: CMIN = 8.445, df = 5 (CMIN/df = 1.689), *p* = 0.133, CFI = 0.974, TLI = 0.949, RMSEA = 0.077 and SRMR = 0.042 (Fig. 1). Monte Carlo simulation analysis indicated that the replication means for CMIN (5.148, s.d. = 3.295), RMSEA (0.028, s.d. = 0.038) and SRMR (0.027, s.d. = 0.009) were less than the original CFA values (online Supplementary Table S2). In this analysis, 10–20% of simulated replication values for CMIN exceeded the original CFA value. Less than 10% of simulated replication values for CMIN/df were under 2, and around 1% of simulated replication values for CMIN/df were <3. In addition, approximately 10% of simulated replication values for RMSEA, and only approximately 5% of simulated replication values for SRMR, exceeded the corresponding original CFA values.

**Table 3.** Results of exploratory factor analysis

				Two-factor model		One-factor model
				1st factor	2nd factor	
*n* = 78		Mean	s.d.	Factor loading	Factor loading	Factor loading
Item #1	I think for myself and make my own life decisions.	3.73	1.30	**0.550**	−0.001	**0.527**
Item #2	I have an idea of what I want to do and/or how I want to be.	3.62	1.35	**0.746**	−0.016	**0.719**
Item #3	I am taking concrete steps to realise what I want to do and/or how I want to be.	3.10	1.26	**0.775**	0.102	**0.713**
Item #4	I express myself in a way that values my own personal style.	2.92	1.36	**0.727**	−0.207	**0.755**
Item #5	I am able to express my thoughts and feelings in my own words.	3.55	1.33	**0.572**	−0.322	**0.641**
Item #6^a^	My choices in lifestyle (e.g. how I use money or time, or my daily life, work) are limited due to things like illness or life circumstances.	2.64	1.43	−0.147	**0.693**	−0.341
Item #7^a^	I give up on doing what I want to do because of my own or others' assumptions that I can't do those things.	3.14	1.38	−0.129	**0.607**	−0.299

Bold indicates the items with factor loadings of 0.4 or higher.

a Items #6 and #7 are reverse-scored.

### Convergent validity and divergent validity

Table 4 shows the results of Pearson's correlation analysis between SPA-5 and other measures. Two participants did not complete the BUES, so the analysis was performed on data from 193 participants. SPA-5 score was significantly and positively correlated with total BUES score (*r* = 0.526, *p* < 0.001), as well as with the subscales ‘Self-esteem-self-efficacy’ (*r* = 0.538, *p* < 0.001), ‘Community activism and autonomy’ (*r* = 0.373, *p* < 0.001) and ‘Optimism and control over the future’ (*r* = 0.336, *p* < 0.001). We found a weak but significant negative correlation between the SPA-5 and the subscale ‘Righteous anger’ (*r* = −0.149, *p* = 0.038). There was no significant correlation between the SPA-5 and the BUES subscale ‘Power-powerlessness’. SPA-5 score was also not correlated with GAF score.

**Table 4.** Results of convergent validity and divergent validity

	Correlation coefficient	*p*-value
Convergent validity (*n* = 193)		
BUES^a^ total	0.526	<0.001
Self-esteem–self-efficacy subscale	0.538	<0.001
Power–powerlessness subscale	0.108	0.134
Community activism and autonomy subscale	0.373	<0.001
Optimism and control over the future subscale	0.336	<0.001
Righteous anger subscale	−0.149	0.038
Divergent validity (*n* = 195)		
Global assessment of functioning	0.001	0.998

a Boston University Empowerment Scale.

### Reliability

In terms of SPA-5 internal consistency, Cronbach's *α* was 0.79. In addition, ICC for test–retest reliability was 0.70.

## Discussion

The present study aimed to develop a brief scale for subjective personal agency that can be completed by people with serious mental illness (online Supplementary file, the final version of the SPA-5). We created the SPA-5 through collaboration with people with schizophrenia and testing of various psychometric properties with ACT service users with schizophrenia.

The item development process and the explanatory factor analysis identified five items for the final SPA. These items appear to encompass the fundamental components of subjective agency as they relate to personal decisions, personal values and individual styles of self-expression, which have been noted in numerous previous studies (Sen, 1985; Alkire, 2005; Ibrahim and Alkire, 2007; Samman and Santos, 2009; Ito and Akimoto, 2015; Tapal *et al*., 2017; Wood and Alsawy, 2018; van Weeghel *et al*., 2019). Compared to a related scale, the Sense of Agency Scale, in which items tend to focus on one's behaviours and ownership of one's actions (Tapal *et al*., 2017), the SPA-5 has a greater focus on the service user's intrapersonal perceptions of their life. The fact that each item statement begins with ‘I’ highlights the subjective focus and nature of the scale. Such item features have been observed in other recent user-led scale development studies, such as the Recovering Quality of Life Scale (Connell *et al*., 2018; Keetharuth *et al*., 2018).

In terms of factor analyses, the two items excluded after EFA were statements related to decision making in a context affected by the individual's health or their circumstances (including their own or others' assumptions). When measuring subjective agency, it may ultimately be suitable to ask about respondents themselves, without regard for the influence of internal or external events. The SPA-5 demonstrated good model fit through CFA (CMIN/df = 1.689, CFI = 0.974, TLI = 0.949, RMSEA = 0.077), according to the COSMIN guidelines for systematic reviews (CFI>0.95, TLI>0.95, RMSEA<0.06 or SRMR<0.08) (Prinsen *et al*., 2018). Even when using more conservative criteria for CFA (good model fit: CMIN/df<2, CFI>0.97, TLI>0.97 and RMSEA<0.05; acceptable model fit: CMIN/df<3, CFI>0.95, TLI>0.95 and RMSEA<0.08) (Schermelleh-Engel *et al*., 2003), the model fit values for the SPA-5 were very close to acceptable. Although the study is based on a relatively small sample, the Monte Carlo simulation analysis found that most simulated replications for CMIN/df, RMSEA and SRMR did not exceed the original CFA values or acceptable criteria values. This suggests that there is a low probability that original CFA values were produced by chance, and appears to confirm the factor validity of the SPA-5.

In terms of convergent validity, overall SPA-5 score was significantly correlated with total BUES score, as well as scores on the subscales ‘Self-esteem-self-efficacy’, ‘Community activism and autonomy’ and ‘Optimism and control over the future’, which are theoretically related to personal agency (Sen, 1985; Alkire, 2005; Ibrahim and Alkire, 2007; Samman and Santos, 2009). At the same time, there was a significant negative correlation between SPA-5 and the subscale ‘Righteous anger’, and no significant correlation between SPA-5 and the subscale ‘Power-powerlessness’. A previous study found that the items in ‘Righteous anger’ and ‘Power-powerlessness’ did not fit well with the Japanese cultural context, in that expressing anger is usually not seen to be the same as expressing oneself (Yamada and Suzuki, 2007). In other words, our results may properly indicate convergent validity of SPA-5 in a Japanese setting, but replication studies are needed in other countries.

In addition, SPA-5 score was not significantly correlated with GAF score. Few studies have directly compared self-rated personal agency and other-rated functioning in people with schizophrenia. In terms of an association between personal recovery and daily functioning, while one meta-analysis found a statistically significant association between self-rated personal recovery scales and GAF in a sample of people with schizophrenia, the correlation was still small (*r* = 0.21) (Van Eck *et al*., 2018). If the results from this meta-analysis can be taken to apply to the relationship between personal agency and functioning in the context of schizophrenia, the fact that no significant correlation between these variables was found in this study may be an indication of the divergent validity of SPA-5. This study also assumed only a relatively small variance in functioning in participants. While the study targeted ACT service users, ACT service agencies generally focus primarily on service users with lower levels of functioning and more severe symptoms than individuals using other outpatient services (Kim *et al*., 2015). Although one meta-analysis found a robust association between schizophrenia symptoms and subjective quality of life among outpatients who are likely to have diverse levels of symptoms (Eack and Newhill, 2007), this meta-analysis and the present study examined different aspects (symptoms *v*. overall functioning) and different outcomes (quality of life *v*. personal agency), and therefore direct comparison is not possible. However, it is possible to say that the characteristics of ACT service users in our sample may have had an impact on the fact that we found no significant correlation between GAF and SPA-5 scores.

The reliability of the SPA-5 was confirmed in our study. COSMIN guidelines for systematic reviews suggest the criteria of Cronbach's *α* of >0.70 for internal consistency, and ICC of >0.70 for test–retest reliability (Prinsen *et al*., 2018). The SPA-5 met these criteria, and appears to have an acceptable level of reliability.

### Strengths and limitations

One of the strengths of this study is that the SPA-5 was developed through collaborative work with people with schizophrenia. As co-production between researchers and service users increases value in the field of health research (Durose *et al*., 2018; Richards *et al*., 2018), it is essential to involve patients in creating patient-reported outcome measures (Staniszewska *et al*., 2012; Wiering *et al*., 2017). The present study included this essential step. In addition, study participants were ACT service users who experience severe symptoms and low levels of functioning in the community. The fact that the SPA-5 can be completed by people with more severe symptoms, and was validated with this population, potentially increases its applicability and usefulness.

Limitations of our study include the sample size, which was the minimum needed for factor analysis, and not relatively large. It is particularly important to note that we used a small subsample when assessing test–retest reliability. A replication study with a larger sample should be performed to confirm our results. In addition, our study did not assess participants' symptoms and cognition. Past meta-analyses have shown that general psychopathology and cognitive function are negatively associated with subjective quality of life in people with schizophrenia (Eack and Newhill, 2007; Tolman and Kurtz, 2012). Subjective perceptions, including subjective feelings of personal agency, may also be influenced by these variables, although our study did not find a relationship between subjective personal agency and overall functioning. A comparison between SPA-5 scores and other objective and subjective measures (e.g. symptoms, cognition and other subjective patient-reported outcome measures, such as quality of life) could produce further evidence of scale validity, and might also contribute to bridging the gap between subjective perceptions and the underlying neuroscience.

While this study has several limitations, the SPA-5 is brief and easy to answer for people with schizophrenia, resulting in a practical patient-reported outcome measure which can be used in research at multiple assessment time points (e.g., cohort study and intervention study), or studies that focus on people with severe mental illness (Yamaguchi *et al*., 2019). In addition, the variables related to subjective personal agency remain unclear in people with severe mental illness, since no validated tools for subjective personal agency in this population had previously existed. SPA-5 can therefore also be used in studies that seek to define and explain the relationships between these variables.

## Conclusion

This study developed a new five-item scale to measure subjective personal agency (the SPA-5) in people with serious mental illness. Factor structure, cross-validity, convergent validity, divergent validity, internal consistency and test–retest reliability were confirmed. Further studies with larger sample sizes, as well as studies outside Japan, should be conducted to confirm these findings and to allow for comparison between the SPA-5 and other relevant objective or subjective variables.

## Supplementary material

For supplementary material accompanying this paper visit http://dx.doi.org/10.1017/S2045796020000256. click here to view supplementary material

## Data

Not all the data are freely accessible because no informed consent was given by the participants for open data sharing, but we can provide the data used in this study to researchers who want to use them, following approval by the ethics committee of the National Center of Neurology and Psychiatry.
