# Supplementary material for: Development and psychometric properties of a new brief scale for subjective personal agency (SPA-5) in people with schizophrenia
Source: Epidemiol Psychiatr Sci. 2020 Apr 10;29:e111. doi: 10.1017/S2045796020000256 (PMC7214545; doi:10.1017/S2045796020000256)
Supplement: Supplementary file 1 [file S2045796020000256sup001.zip › Supplementary_material/Supplementary_File_final_ver_SPA5_English.docx]

**Questionnaire form: English version**

**Five-item Subjective Personal Agency scale (SPA-5)**

| Please tell us how you currently think or feel about the following items. For each item, select the most applicable response, and place a check mark (✓) in the corresponding box (□). | | | | | | |
| --- | --- | --- | --- | --- | --- | --- |
|  | | Strongly disagree | Disagree | Neither agree nor disagree | Agree | Strongly Agree |
| 1 | I think for myself and make my own life decisions. | □ | □ | □ | □ | □ |
| 2 | I have an idea of what I want to do and/or how I want to be. | □ | □ | □ | □ | □ |
| 3 | I am taking concrete steps to realize what I want to do and/or how I want to be. | □ | □ | □ | □ | □ |
| 4 | I express myself in a way that values my own personal style. | □ | □ | □ | □ | □ |
| 5 | I am able to express my thoughts and feelings in my own words. | □ | □ | □ | □ | □ |

- Scoring: Strongly disagree = 1, Disagree = 2, Neither agree nor disagree = 3, Agree = 4, Strongly agree = 5
- Total score is calculated by summing individual item scores.
- Translation from Japanese into English was conducted by a co-author (PB), and back-translation from English into Japanese was conducted by a Japanese research staff member who obtained her master’s degree in the USA and is fluent in English.
- You are free to use the SPA-5 without restriction, but acknowledgement of the source would be appreciated.
