# Supplementary material for: Development and psychometric properties of a new brief scale for subjective personal agency (SPA-5) in people with schizophrenia
Source: Epidemiol Psychiatr Sci. 2020 Apr 10;29:e111. doi: 10.1017/S2045796020000256 (PMC7214545; doi:10.1017/S2045796020000256)
Supplement: Supplementary file 1 [file S2045796020000256sup001.zip › Supplementary_material/Supplementary_File_final_ver_SPA5_Japanese.docx]

**Questionnaire form: Japanese version**

**Five-item Subjective Personal Agency scale (SPA-5)：5項目版主観的主体性尺度**

| 現在のあなたの状況について教えてください。  あてはまるものを１つ選び、□の中に✓をつけてください。 | | | | | | |
| --- | --- | --- | --- | --- | --- | --- |
|  | | そう思わない | あまり  そう思わない | どちらとも  いえない | ややそう思う | そう思う |
| 1 | 私は、人生で何か決めるときは、自分で考えて決めている | □ | □ | □ | □ | □ |
| 2 | 私には、「やりたいこと」や「こうありたい」というイメージがある | □ | □ | □ | □ | □ |
| 3 | 私は、「やりたいこと」や「こうありたい」ことのために、具体的に何かしている | □ | □ | □ | □ | □ |
| 4 | 私は、自分のスタイルを活かして、自分を表現している | □ | □ | □ | □ | □ |
| 5 | 私は、自分の言葉で思いを表現している | □ | □ | □ | □ | □ |

- 得点: 「そう思わない」 = 1, 「あまりそう思わない」 = 2, 「どちらともいえない」 = 3,

「ややそう思う」 = 4, 「そう思う」 = 5

- 合計得点：それぞれの項目の得点を足し上げる

- SPA-5は、適切な引用をしていただければ、著者に連絡することなく利用可能です。
