# Supplementary material for: Development and psychometric properties of a new brief scale for subjective personal agency (SPA-5) in people with schizophrenia
Source: Epidemiol Psychiatr Sci. 2020 Apr 10;29:e111. doi: 10.1017/S2045796020000256 (PMC7214545; doi:10.1017/S2045796020000256)
Supplement: Supplementary file 1 [file S2045796020000256sup001.zip › Supplementary_material/Supplementary_Tables_and_Figs.docx]

**Online Supplementary Table S1. Initial seven items considered for Subjective Personal Agency scale**

|  | | | | | | |
| --- | --- | --- | --- | --- | --- | --- |
|  | | ***Strongly Disagree*** | ***Disagree*** | ***Neither agree nor disagree*** | ***Agree*** | ***Strongly Agree*** |
| 1 | I think for myself and make my own life decisions. | □ | □ | □ | □ | □ |
| 2 | I have an idea of what I want to do and/or how I want to be. | □ | □ | □ | □ | □ |
| 3 | I am taking concrete steps to realize what I want to do and/or how I want to be. | □ | □ | □ | □ | □ |
| 4 | I express myself in a way that values my own personal style. | □ | □ | □ | □ | □ |
| 5 | I am able to express my thoughts and feelings in my own words. | □ | □ | □ | □ | □ |
| 6 | My choices in lifestyle (for example, how I use money or time, or my daily life, work) are limited due to things like illness or life circumstances. | □ | □ | □ | □ | □ |
| 7 | I give up on doing what I want to do because of my own or others’ assumptions that I can’t do those things. | □ | □ | □ | □ | □ |

Items #6 and #7 are reverse-scored.

**Online Supplementary Figure S1. Participant recruitment process**

**
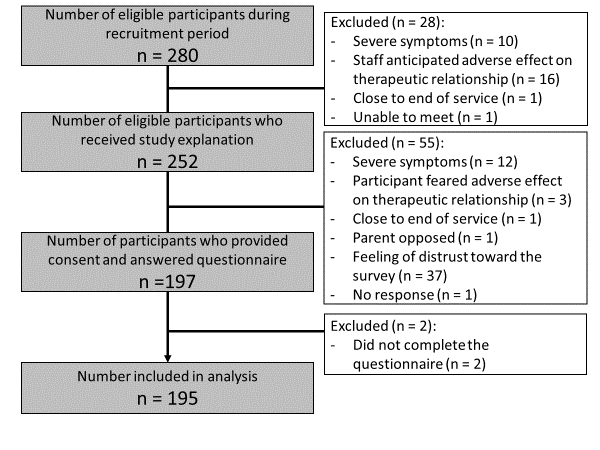
**

**Online Supplementary Figure S2. Scree plot from exploratory factor analysis for Subjective Personal Agency scale**

**
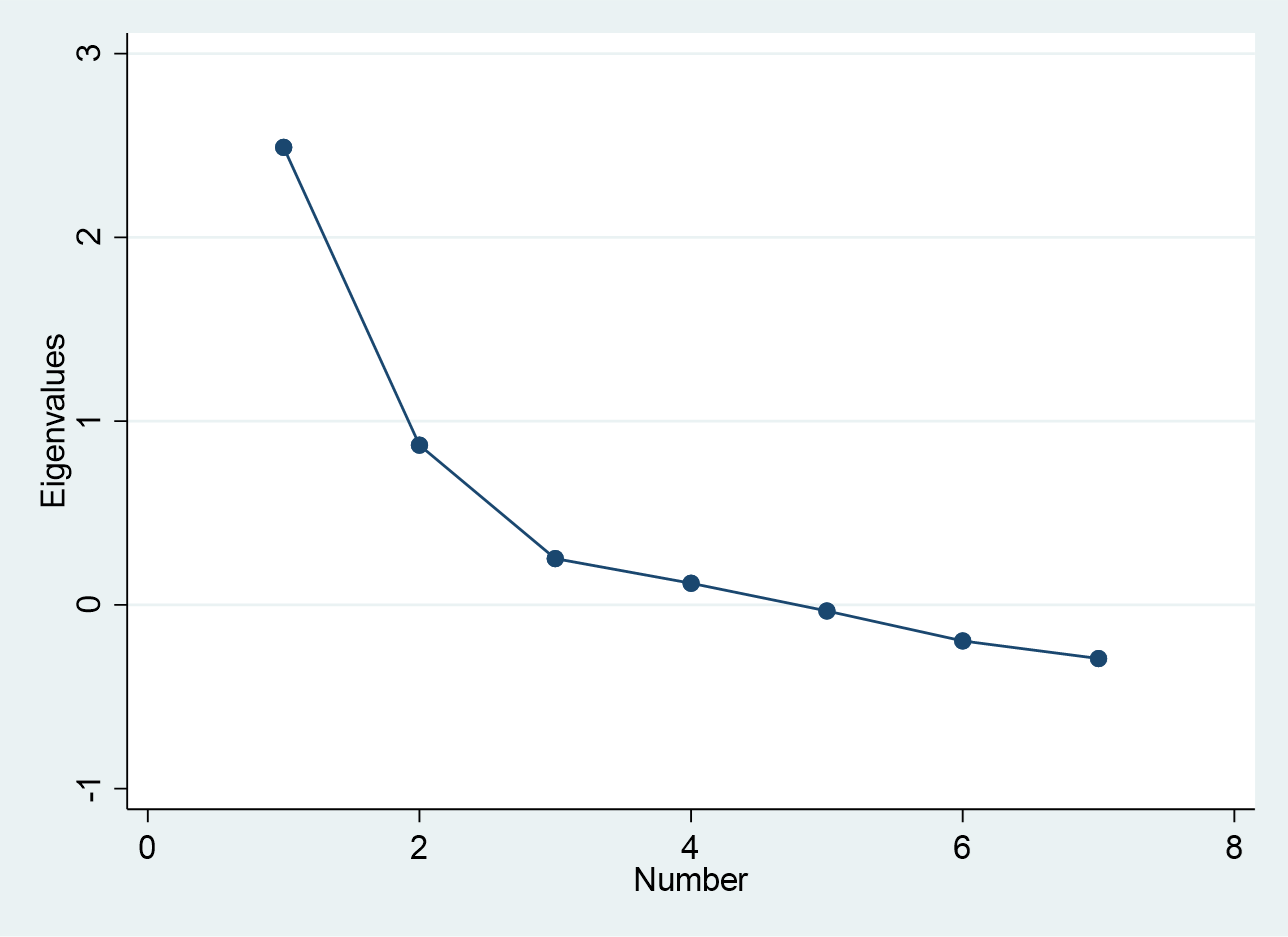
**

**Online Supplementary Table S2. Results of Monte Carlo simulation analysis**

| CMIN^1^ | | | | RMSEA^2^ | | | | SRMR^3^ | | | |
| --- | --- | --- | --- | --- | --- | --- | --- | --- | --- | --- | --- |
| Proportions | | Percentiles | | Proportions | | Percentiles | | Proportions | | Percentiles | |
| Expected | Observed | Expected | Observed | Expected | Observed | Expected | Observed | Expected | Observed | Expected | Observed |
| 0.990 | 0.990 | 0.554 | 0.553 | 0.990 | 1.000 | -0.061 | 0.000 | 0.990 | 0.999 | 0.006 | 0.009 |
| 0.980 | 0.980 | 0.752 | 0.754 | 0.980 | 1.000 | -0.051 | 0.000 | 0.980 | 0.993 | 0.008 | 0.010 |
| 0.950 | 0.955 | 1.145 | 1.208 | 0.950 | 1.000 | -0.035 | 0.000 | 0.950 | 0.967 | 0.012 | 0.013 |
| 0.900 | 0.908 | 1.610 | 1.675 | 0.900 | 1.000 | -0.021 | 0.000 | 0.900 | 0.911 | 0.015 | 0.016 |
| 0.800 | 0.810 | 2.343 | 2.405 | 0.800 | 1.000 | -0.004 | 0.000 | 0.800 | 0.796 | 0.019 | 0.019 |
| 0.700 | 0.712 | 3.000 | 3.070 | 0.700 | 0.432 | 0.008 | 0.000 | 0.700 | 0.675 | 0.022 | 0.021 |
| 0.500 | 0.520 | 4.351 | 4.491 | 0.500 | 0.382 | 0.028 | 0.000 | 0.500 | 0.470 | 0.027 | 0.026 |
| 0.300 | 0.311 | 6.064 | 6.170 | 0.300 | 0.285 | 0.048 | 0.045 | 0.300 | 0.283 | 0.031 | 0.031 |
| **0.200** | 0.208 | **7.289** | 7.409 | 0.200 | 0.221 | 0.060 | 0.064 | 0.200 | 0.190 | 0.034 | 0.034 |
| **0.100** | 0.106 | **9.236** | 9.369 | **0.100** | 0.142 | **0.077** | 0.086 | 0.100 | 0.105 | 0.038 | 0.038 |
| 0.050 | 0.056 | 11.070 | 11.342 | 0.050 | 0.086 | 0.091 | 0.104 | **0.050** | 0.059 | **0.041** | 0.042 |
| 0.020 | 0.025 | 13.388 | 14.059 | 0.020 | 0.046 | 0.107 | 0.124 | 0.020 | 0.030 | 0.045 | 0.046 |
| 0.010 | 0.015 | 15.086 | 16.016 | 0.010 | 0.028 | 0.117 | 0.137 | 0.010 | 0.017 | 0.047 | 0.050 |
| **Monte Carlo simulation analysis, summary** | | | | | | | | | | | |
| Mean | 5.148 |  |  | Mean | 0.028 |  |  | Mean | 0.027 |  |  |
| SD | 3.295 |  |  | SD | 0.038 |  |  | SD | 0.009 |  |  |

CMIN: chi-squared statistic; RMSEA: root mean square error of approximation; SRMR: standardized root mean squared residuals

1. Original CFA value for CMIN = 8.445
2. Original CFA value for RMSEA = 0.077
3. Original CFA value for SRMR = 0.042
